# NFAT1 Is Highly Expressed in, and Regulates the Invasion of, Glioblastoma Multiforme Cells

**DOI:** 10.1371/journal.pone.0066008

**Published:** 2013-06-06

**Authors:** Xinxin Tie, Sheng Han, Lingxuan Meng, Yunjie Wang, Anhua Wu

**Affiliations:** Department of Neurosurgery, The First Hospital of China Medical University, Shenyang, China; Beijing Tiantan Hospital, Capital Medical University, China

## Abstract

Members of the nuclear factor of activated T cells (NFAT) family have been identified as regulators of oncogenic transformation in several human malignancies. A prominent member of this family, NFAT1, is associated with tumor cell survival, apoptosis, migration and invasion. Here, we investigated the role of NFAT1 in glioma cells. In 111 clinical samples, microarray analysis demonstrated that NFAT1 was over-expressed in glioblastoma multiforme (GBM), compared with low-grade gliomas, a result confirmed by RT-PCR in 24 clinical samples and in the U87 and U251 cell lines. Immunohistochemistry and immunofluorescence stain indicated that over-expressed NFAT1 was mainly located in the nucleus, where it acted as a transcription factor. After treatment with the NFAT antagonist cyclosporin A (CsA) and FK506, levels of NFAT1 in the nuclei of U87 GBM cells were dramatically reduced. The invasive potential of U87 cells was reduced by the same treatment, as well as by inhibition of NFAT1 expression using small hairpin RNA. Proliferation of U87 cells was unaffected by CsA, FK506 and NFAT1 shRNA transfection. Clustering analysis and Pearson correlation analysis of microarray data showed that the expression of NFAT1 correlated with the expression of the invasion-related genes cyclooxygenase-2 (COX-2), matrix metalloproteinase-7 (MMP-7) and MMP-9, a result confirmed by *in vitro* analysis. These findings demonstrate that NFAT1 contributes to the invasive potential but not the proliferation of GBM cells, and suggest that CsA may find application as an adjuvant in combined treatment strategies for GBM.

## Introduction

Glioma is the most common primary brain tumor and glioblastoma multiforme (GBM) is the most aggressive type of glioma [1]. In spite of surgery, chemotherapy and radiation, GBM quickly invades healthy brain tissue, with a median survival time of about 14 months [2], and the highly invasive nature of GBM cells is thought to contribute to the poor prognosis of this tumor. Many factors are involved in the migration and invasion of malignant tumors [3], [4], [5], [6], [7], [8], of which NFATs are of special interest since they play key roles in the activation and differentiation of T cells. We have proposed that the NFAT signaling pathway regulates invasion or proliferation of malignant cells with a mechanism similar to T cells. A critical corollary of this hypothesis is that any treatment strategy targeting the NFAT pathway may also affect the T cell function, which may lead to failure of anti-tumor immunotherapy.

In our previous study, we demonstrated that NFAT1 is involved in overexpression of interleukin-13 receptor alpha2 subunit (IL-13Ra2) in GBM [9]. Due to the elevated expression of IL-13Ra2 in GBMs, we hypothesized that NFAT1 would be similarly highly expressed and activated in GBM. NFAT1 (NFATc2) is the prevalent NFAT family member expressed in peripheral T lymphocytes and many other cells outside the immune system. In the steady state, NFAT1 is a heavily phosphorylated protein that is dephosphorylated and activated by the phosphatase calcineurin, which is the molecular target of the immunosuppressive agent cyclosporin A (CsA) and FK506 (tacrolimus). Dephosphorylation of NFAT1 results in nuclear translocation, and the subsequent initiation of specific transcriptional programs [10]. In this study, we show for the first time that NFAT1 is overexpressed and activated in GBM, and that NFAT1 contributes to the invasion but not proliferation of GBM. Furthermore we demonstrate that the effects of NFAT1 may be mediated by induction of cyclooxygenase-2 (COX-2), matrix metalloproteinase-7 (MMP-7) and MMP-9 expression.

## Materials and Methods

### Patients and Samples

One hundred and thirty-five clinical samples were collected from the Chinese Glioma Genome Atlas, including 90 primary GBMs (P), 8 anaplastic astrocytomas (AA) and 37 astrocytomas (A). All patients underwent surgical resection between January 2005 and December 2009. The histological diagnosis was established and verified by two neuropathologists according to the 2007 World Health Organization (WHO) classification guidelines. This study was approved by the institutional review boards of all participating hospitals, and written informed consent was obtained from every patient.

### Microarray Analysis

The 135 clinical samples were immediately snap-frozen in liquid nitrogen after resection. For each sample, prior to RNA extraction the percentage of tumor cells was assessed using a hematoxylin and eosin–stained frozen section. Only samples with more than 80% tumor cells were selected for analysis. The mirVana miRNA Isolation kit (Ambion) was used for total RNA extraction, according to the manufacturer’s protocol. RNA concentration and quality were measured using a NanoDrop ND-1000 spectrophotometer (NanoDrop Technologies). 111 samples (66P, 8AA, 37A) went forward to microarray analysis using Agilent Whole Human Genome Arrays according to the manufacturer’s instructions. The integrity of total RNA was surveyed using an Agilent 2100 Bioanalyzer. Complementary DNA and biotinylated cRNA was synthesized and hybridized to the array. An Agilent G2565BA Microarray Scanner System and Agilent Feature Extraction Software (version 9.1) were used for data acquisition. Probe intensities were normalized using GeneSpring GX 11.0 (Agilent).

### Cell Culture

The human GBM cell lines U87-MG and U251 were obtained from the Chinese Academy of Sciences Cell Bank (Shanghai, China) and maintained in Dulbecco’s Modified Eagle’s Medium (DMEM) supplemented with 10% fetal bovine serum (Invitrogen). Cells were incubated at 37°C with 5% CO_2_.

### RT-PCR

Total RNA was isolated from U87, U87-control-shRNA, U87-NFAT1-shRNAand U251 cell lines using TRIzol reagent (Invitrogen) according to the manufacturer's protocol. Total RNA from U87, U87-NFAT1-shRNA, U251 cell lines and 24 clinical primary GBM samples was reverse transcribed into cDNA and used for PCR amplification. Specific primers for NFAT1, COX-2, MMP-7, MMP-9 and GAPDH were: NFAT1 forward: 5′-CGG GCC CAC TAT GAG ACA GAA-3′ and NFAT1 reverse: 5′-GCT CAT CAG CTG TCC CAA TGA A-3′; COX-2 forward: 5′-CAA AAG CTG GGA AGC CTT CTC TAA-3′ and COX-2 reverse: 5′-GCC CAG CCC GTT GGT GAA AG-3′ [11]; MMP-7 forward: 5′-AAA CTC CCG CGT CAT AGA AAT-3′ and MMP-7 reverse: 5′-TCC CTA GAC TGC TAC CAT CCG-3′ [12]; MMP-9 forward 5′-CAA ACC CTG CGT ATT TCC-3′: and MMP-9 reverse 5′-AGA GTA CTG CTT GCC CAG GA-3′ [13]. GAPDH forward: 5′-GCA CCG TCA AGG CTG AGA AC-3′ and GAPDH reverse: 5′-TGG TGA AGA CGC CAG TGG A-3′ (TaKaRa) respectively. RT-PCR reactions were repeated at least three times.

### Immunohistochemistry

From the 135 clinical samples, 50 samples (25P and 25A) were randomly selected for immunohistochemistry staining. All paraffin-embedded sections were deparaffinized followed by washing in xylenes and serial dilutions of ethanol. Endogenous peroxidase was blocked by 3% H_2_O_2_ for 12 min. After antigen retrieval, blocks for avidin and biotin and Fc receptor were applied. A primary antibody against NFAT1 (Abcam, Cambridge, UK; 1∶50) was applied and incubated overnight at 4°C. Antibody binding was visualized using a biotinylated secondary antibody, avidine-conjugated peroxidase (ABC method; Vector Laboratories), and 3,3’ diaminobenzidine tetrachloride as a chromogen, and hematoxylin as a counterstain.

### Immunofluorescence

U87 cells grown on coverslips were left untreated or treated with CsA (1 µg/ml) or FK506 (100 ng/mg) for 60 min. Cells were then washed, fixed, blocked and probed with anti-NFAT1 antibody (Abcam, Cambridge, UK; 1∶100). NFAT1 was detected with a fluorochrome-conjugated secondary antibody and nuclei were counterstained with Hoechest 33342. Coverslips were mounted on glass slides and cells were observed with a confocal microscope (Olympus FV1000S-SIM, Japan).

### NFAT1 Gene Expression Knockdown

NFAT1 small hairpin RNA (shRNA) plasmid and control shRNA plasmid (Santa Cruz Biotechnology) were transfected into U87 cells according to the manufacturer's protocol. U87 cells were seeded in a six well plate and grown to 50–70% confluency in antibiotic-free DMEM supplemented with 10% FBS. The cells were washed twice with 2 ml of shRNA Transfection Medium (Santa Cruz Biotechnology) and then 0.8 ml of shRNA Plasmid Transfection Medium was added. After adding 200 µl shRNA Plasmid DNA/shRNA Plasmid Transfection Reagent Complex (Santa Cruz Biotechnology), the cells were incubated for 8 h at 37°C with 5% CO_2._ One milliliter of DMEM with 20% FBS was added. Forty-eight hours post-transfection, the medium was replaced with fresh medium containing 5 µg/ml puromycin for selection of stably transfected cells. Medium was changed every 2 days. Four days later, NFAT1 gene expression was monitored using western blot analysis.

Cells were washed with PBS and lysed using ice-cold lysis buffer. The protein was separated by SDS–PAGE and transferred onto nitrocellulose membranes followed by incubation in blocking solution for 2 h at room temperature. Membranes were then washed and incubated with monoclonal antibodies against NFAT1 (Abcam, Cambridge, UK; 1:1000) followed by incubation with alkaline phosphatase-labeled secondary anybody. Bands were detected using a chemiluminescence ECL kit (Santa Cruz Biotechnology).

### Invasion Assay

Eight-micrometer pore transwell chambers (Corning) were coated with 50 µl Matrigel (BD Bioscience). 2×10^3^ cells (untreated U87, U87 treated with CsA or FK506, U87-NFAT1-shRNA and U87-control-shRNA) were resuspended in 100 µl serum-free DMEM containing 0.1% bovine serum albumin and added in triplicate to transwell chambers. DMEM with 20% FBS (600 µl) was added to the bottom chamber. Cells were allowed to invade the Matrigel-coated filters toward the lower compartment for 20 h at 37°C. Cells that had invaded to the lower surface of the filter were fixed and stained, and the number of cells was counted under microscope. A total of ten fields were counted for each transwell filter.

### MTT Assay

The MTT [3-(4,5-dimethylthiazolyl-2)-2,5-diphenyltetrazolium bromide] assay was performed to detect cell proliferation. Briefly, U87 cells were seeded in 96-well plates at a density of 2×10^3^/well. After 24 h of incubation, cells were serum starved overnight. Cells were left untreated or treated with CsA or FK506 for 24, 48, 72, 96 or 120 h. In another experiment, U87-NFAT1-shRNA and U87-control-shRNA cells were seeded in 96-well plates at a density of 2×10^3^/well and incubated for 24, 48, 72, 96 or 120 h. At each time point, 20 µl of 5 mg/ml MTT solution was added to each well. After 4 h of incubation, medium was removed from the wells by aspiration and formazan crystals were dissolved in 150 µl of dimethyl sulfoxide (DMSO). Color intensity was measured at 490 nm with an enzyme linked immunosorbent assay plate reader (Tecan Sunrise Remote, Austria).

### Cell Cycle Analysis

To assess the cell cycle, U87-control-shRNA and U87-NFAT1-shRNA cells were separately plated in six-well microtiter plates. After 24 h, the cells were trypsinized and washed once with PBS. The cells were then stained with propidium iodide (PI, 75 µM) in the presence of NP-40. Analysis of the DNA content was performed by collecting 10,000 events for cell cycle analysis using a FACScalibur flow cytometer and CellQuest software (BD Biosciences, San Jose, CA, USA).

### Cluster Analysis and Statistical Analysis

The mRNA expression of NFAT1, COX-2, MMP-2, MMP-7, MMP-9 and integrinβ4 obtained from microarray analysis (http://jzlcn.dajiankang.com/portal.php) were used for cluster analysis (Cluster 2.20) and Pearson correlation analysis (SPSS 13.0). Cluster analysis was performed using the hierarchical clustering method with average linkage. The results of cluster analysis were visualized using TreeView software. Chi-square test and Student’s t test were used to determine significant differences. All data are presented as the mean ±standard error of three independent experiments. A two-tailed P-value of <0.05 was regarded as statistically significant.

## Results

### NFAT1 is Overexpressed in GBM Samples and Cell Lines

**Figure 1:**
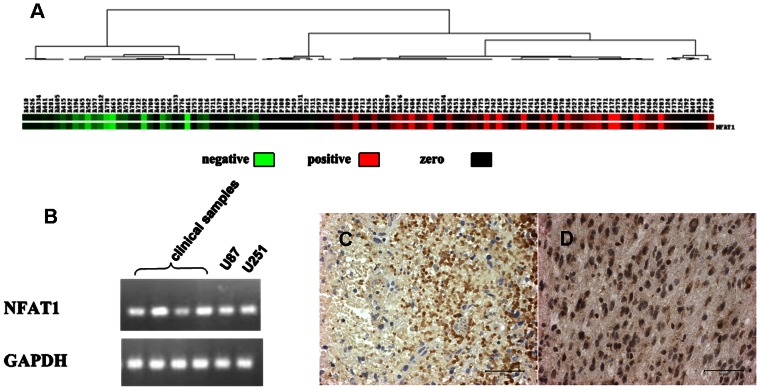
Expression of NFAT1 in glioma. A: The mRNA expression of NFAT1 obtained from microarray analysis in 111 clinical samples was analyzed by cluster analysis and visualized by Treeview software. Compared with low-grade glioma, NFAT1 was overexpressed in GBM. B: RT-PCR was used to examine the mRNA expression of NFAT1 in U87, U251 GBM cell lines and 24 clinical GBM samples. Confirming the result of microarray analysis, high expression of NFAT1 was detected in all the samples. C: NFAT1 expression and location examined by immunohistochemistry in GBM and astrocytoma (40×). The deep brown NFAT1 staining was mainly located in the nuclei of GBM cells, and the cytoplasm of cells was also stained brown. D: Only a few astrocytoma cells stained light brown in the cytoplasm.

To investigate whether NFAT1 was up-regulated at the mRNA level in GBMs, we chose 111 clinical samples of different histological types (66P, 8AA, 37A) for microarray analysis. The microarray analysis established that NFAT1 was highly expressed in GBMs, compared with low-grade gliomas (**Fig. 1A** **)**. Next, RT-PCR was used to measure NFAT1 levels in two GBM cell lines (U87 and U251) and 24 GBM clinical samples. Confirming the findings of microarray analysis, RT-PCR showed high expression of NFAT1 in all the GBM cell lines and clinical samples (**Fig. 1B**).

### Overexpressed NFAT1 is Activated in GBMs and Repressible by CsA and FK506

**Figure 2:**
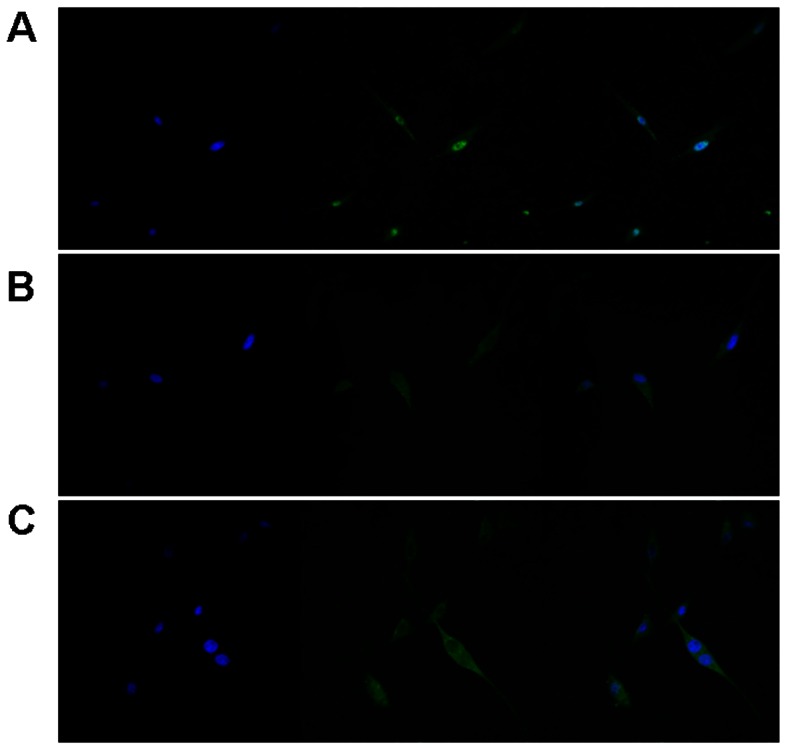
Immunofluorescence stain detection of NFAT1 localization in U87 cells. U87 cells were untreated (A) or treated with CsA (1 µg/ml) or FK506 (100 ng/mg) for 60 min. CsA or FK506 caused NFAT1 (green signals) translocation from nucleus (blue signals) to cytoplasm (B and C).

Since only NFAT1 functions as a transcription factor only in the nucleus, we next examined the expression and intracellular location of NFAT1 at protein level. In 50 clinical samples (25P and 25A), immunohistochemistry stain showed that NFAT1 protein was overexpressed and mainly located in the nuclei in GBM cells (23/25), while in astrocytomas NFAT1 was expressed at a low level and confined to the cytoplasm (6/25) (**Fig. 1C**). In addition, immunofluorescence stain detected that in U87 cells, NFAT1 was also mainly located in the nuclei. Similar to other cells, treatment of U87 cells with the calcineurin inhibitors CsA or FK506 caused rapid inhibition and nuclear export of NFAT1 (**Fig. 2**).

### NFAT1 Inhibition or Down-regulation Reduces Cell Invasion in GBM Cells

**Figure 3:**
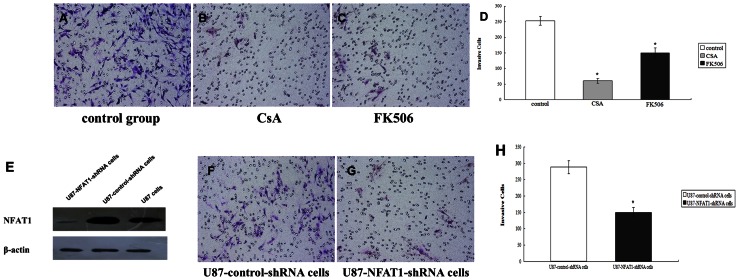
Effect of NFAT1 inhibition on invasion of GBM U87 cells. U87 cells were untreated or treated with CsA or FK506. The microscopic image of the invasive cells is shown to the left (A, B and C). The mean values from duplicate samples of three independent experiments are shown to the right (D). E: Western blot analysis was used to examine the expression of NFAT1 in U87, U87-control-shRNA and U87-NFAT1-shRNA cells. NFAT1 expression was stably knocked down in U87-NFAT1-shRNA cells. (F-H) Effect of NFAT1 down-regulation on invasion of GBM U87 cells. The microscopic image of the invasive cells is shown to the left (F and G). The mean values from duplicate samples of three independent experiments are shown to the right (H). *, P<0.05.

Because NFAT1 is overexpressed and activated in GBMs, we asked whether NFAT1 plays a role in the malignant phenotype of GBM. First of all, to evaluate the effect of NFAT1 on cell invasion, we performed a Matrigel invasion assay in U87 cells. As shown in **Fig. 3A**–**D**, the invasiveness of U87 cells was significantly reduced by treatment with the NFAT1 inhibitors CsA or FK506.

Next, NFAT1 was stably knocked down in U87 cells by transfection of NFAT1-specific shRNA plasmid. The loss of NFAT1 expression was confirmed by western blot analysis with anti-NFAT1 (**Fig. 3E**). Down-regulation of NFAT1 in U87-NFAT1-shRNA cells resulted in a significant reduction in cell invasion compared with cells transfected with non-silencing control shRNA plasmid (**Fig. 3F**–**H**). Together, these data suggest that under normal conditions NFAT1 contributes to the invasive phenotype of GBM.

### NFAT1 Inhibition or Down-regulation has no Effect on Cell Proliferation in GBM Cells

**Figure 4:**
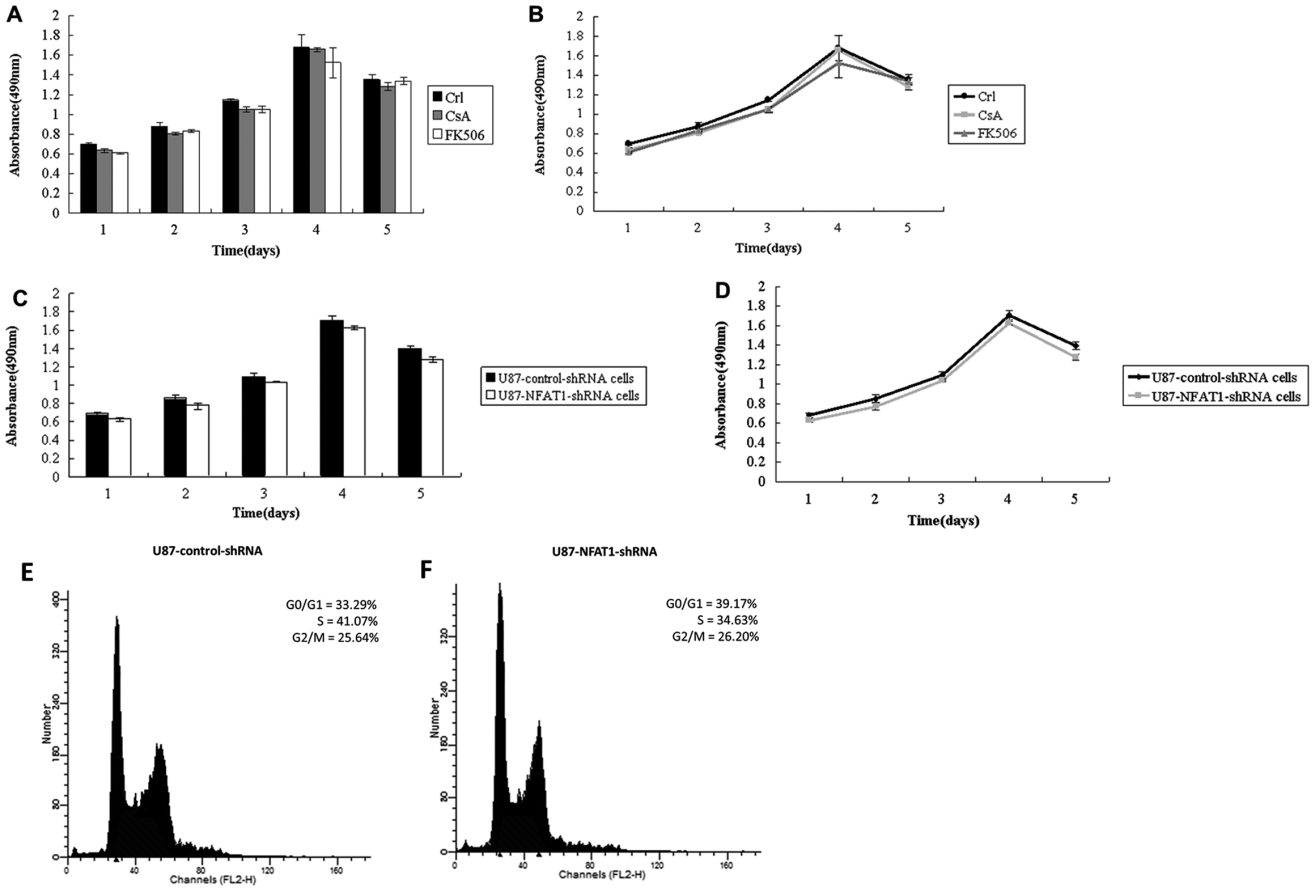
Effect of NFAT1 inhibition by CsA or FK506 on proliferation of GBM U87 cells (A and B). Effect of NFAT1 down-regulation on proliferation of GBM U87 cells (C and D). The cells were plated in triplicate. The graphs are representative of three independent experiments. E-F: Cell cycle was analyzed by the incorporation of PI. The cells were seeded in triplicate and analyzed 24 h after plating by flow cytometry. The percentage of cells in each phase of the cell cycle is indicated in the graphs. Down-regulation of NFAT1 had no detectable effect on the cell cycle of U87 cells.

We next assessed the role of NFAT1 in cell proliferation using an MTT assay. As shown in **Fig. 4A**–**B**, the proliferation of U87 cells was not significantly affected by blocking the activation of NFAT1 with inhibitors CsA or FK506 at any tested time point. Similarly, comparing U87-NFAT1-shRNA cells with U87-control-shRNA cells, knockdown of NFAT1 expression by stable transfection with NFAT1-specific shRNA plasmid had no detectable effect on cell proliferation (**Fig. 4C**–**D**). Next, to assess the effect of NFAT1 down-regulation on the number of cells entering the cell cycle, we used PI staining to evaluate the cell cycle profile of U87-control-shRNA and U87-NFAT1-shRNA cells 24 h after plating. Cell cycle analysis showed that down-regulation of NFAT1 had no detectable effect on the cell cycle of U87 cells. Collectively, these results indicate that activated NFAT1 does not regulate cell proliferation in GBM.

### Expression of NFAT1 is Correlated with the Expression of COX-2, MMP-7 and MMP-9 in GBMs

**Figure 5:**
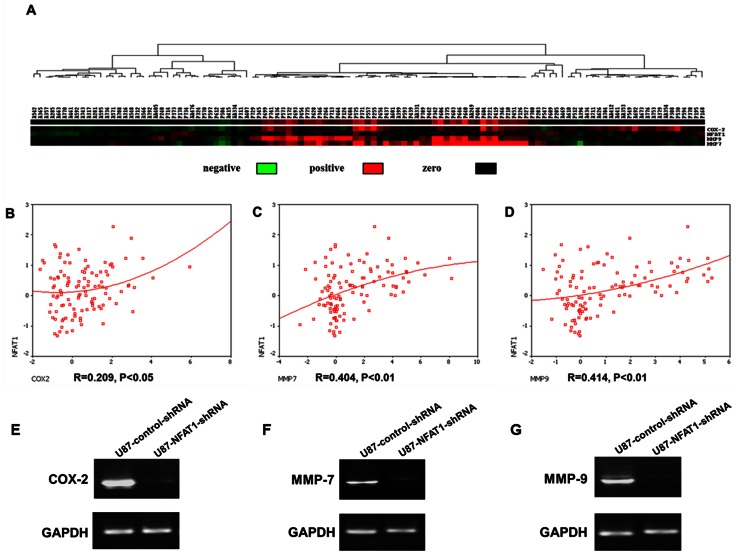
The mRNA expression of NFAT1, COX-2, MMP-7 and MMP-9 obtained from microarray analysis in 111 clinical samples was analyzed by cluster analysis and Pearson correlation analysis. (A) Cluster Treeview showed that high co-expression of NFAT1, COX-2, MMP-7 and MMP-9 was only found in GBM samples. (B, C and D) Correlation between the expression of NFAT1 and the expression of COX-2, MMP7 and MMP-9 E-F: RT-PCR demonstrated that NFAT1 knockdown by specific shRNA resulted in a significant reduction of COX-2, MMP-7 and MMP-9 mRNA expression.

We next set out to characterize the target genes induced by NFAT1 that contribute to promoting invasion in GBMs. mRNA expression levels of NFAT1, COX-2, MMP-2, MMP-7, MMP-9 and integrinβ4 obtained from microarray analysis in 111 clinical samples were analyzed with cluster analysis and Pearson correlation analysis. The expression of NFAT1 was significantly correlated with that of COX-2 (R = 0.209, P<0.05), MMP-7(R = 0.404, P<0.01) and MMP-9 (R = 0.414, P<0.01) in gliomas. Moreover NFAT1, COX-2, MMP-7 and MMP-9 were simultaneously highly expressed in GBM, but not in low-grade gliomas (**Fig. 5A**–**D**). Using RT-PCR, we next observed that NFAT1 knockdown by specific shRNA caused a significant reduction in the mRNA levels of COX-2, MMP-7 and MMP-9 (**Fig. 5E**–**F**). These results demonstrate that the expression of COX-2, MMP-7 and MMP-9 is induced by NFAT1, and indicate that NFAT1 may promote invasion through induction of these genes.

## Discussion

NFAT1 was initially identified as a transcriptional regulator with profound effects on the proliferation and migration of T cells [14]. Over the last few years, numerous studies have reported abnormal NFAT1 signaling, overexpression and inappropriate activation in tumor development and metastasis [14], [15], [16]. It is conceivable therefore that NFAT1 may, under some conditions, play a similar role in T cells and tumor cells, and particularly in the highly invasive and proliferative GBM [17], [18], [19], [20], [21], [22], [23], [24], [25], [26], [27], given that the immune system plays a significant role in the formation and development of glioma. By extension, manipulation of NFAT1 signaling may potentially affect the extent of the GBM-related immune response. Identifying the exact role of NFAT1 in tumor cells can pave the way to glioma specific immunotherapy and, at the same time, avoid increasing the invasion and proliferation of glioma cells.

We discovered that NFAT1 is essential for the high level expression of IL-13Ra2 in GBM [9], and accordingly hypothesized that NFAT1 was highly expressed and active in GBM. To date, there have been no reports investigating the role of NFAT1 in glioma. In the current study, using whole genome gene profiling in 111 clinical samples, we found that NFAT1 expression was increased in GBM compared with astrocytoma. We confirmed this finding in primary GBM tissues and GBM cell lines using RT-PCR. Both immunohistochemistry and immunofluorescence staining showed overexpression and nuclear localization of NFAT1 protein in GBM cells. Inhibition of calcineurin activity by CsA or FK506 caused rapid inactivation and nuclear export of NFAT1 in U87 cells. Collectively, these data suggest that NFAT1 signaling is highly active in GBM cells. Moreover, we demonstrated that NFAT1 is activated not only in vivo but also long term cultured glioma cell lines, suggesting that activation of NFAT1 may not be affected by tumor microenvironment and is an inherent characteristic of GBM.

We proceeded to explore the role of NFAT1 in GBM. NFAT1 was highly expressed in all the glioma cell lines we examined. We randomly chose U87 cells for further examination. Specific inhibitors of NFAT1 pathways or NFAT1 shRNA were used to knockdown the expression of NFAT1 in U87 cells. Using in vitro invasion assay, we found that blocking the activation of NFAT1 with CsA or FK506 significantly decreased the invasiveness of U87 cells. Since the inhibitory effect of CsA and FK506 is not specific, we developed clones of U87-NFAT1-shRNA cells in which NFAT1 expression is stably knocked down by NFAT1-specific shRNA and showed that the invasive ability of these cells was markedly reduced. Although there are many methods to measure invasion ability of cells, our trans-well assay clearly showed that NFAT1 contributes to the invasive phenotype of GBM. We next characterized the genes which are involved in the promotion of GBM cell invasion by NFAT1 signaling. Induction of COX-2 by NFAT1 has been reported in T lymphocytes, vascular smooth muscle cells and other nonimmune cells [28], [29]. Moreover, previous studies have provided evidence that COX-2 expression is induced by NFAT1 in colon carcinoma and breast cancer cells [15], [30]. Furthermore, it has been reported that NFAT1 is target of α6β4 integrin signaling and is involved in promoting carcinoma invasion [16], and that it contributes to induction of MMPs [16]. Given that COX-2, MMP-2, MMP-7, MMP-9 and integrinβ4 are all critical for inducing an invasive phenotype in malignant cells [31], and have been linked to NFAT1, the mRNA expression of these genes was analyzed by microarray analysis of clinical samples. The results showed that expression of NFAT1 was significantly correlated with the expression of COX-2, MMP-7 and MMP-9. Furthermore, elevated co-expression of NFAT1, COX-2, MMP-7 and MMP-9 was restricted to GBM samples. These results were confirmed by our in vitro analysis, which demonstrated that specific knockdown of NFAT1 in U87 cells led to marked reduction of COX-2, MMP-7 and MMP-9 expression. We therefore proposed that the likely targets for NFAT1 in inducing an invasive phenotype in GBM are COX-2, MMP-7 and MMP-9.

Unexpectedly, both MTT and cell cycle assays showed that inhibition or specific down-regulation of NFAT1 had no detectable effect on the proliferation of, or the cell cycle in, GBM cells. These results are inconsistent with studies showing that overexpression of NFAT1 suppresses proliferation of T47D cells [32], and that constitutive expression of NFAT1 induces apoptosis in NIH3T3 fibroblasts [33], [34]. In contrast, up-regulation of NFAT1 expression promotes cell cycle progression and proliferation of intrahepatic cholangiocarcinoma cells [35]. These results may reflect the possibility that NFAT1 plays different roles in different tissues and cells. Unlike our study, in these studies, expression of NFAT1 was up-regulated or over-activated from the normal level, which may activate different signaling pathways and resulted in different patterns of cell transformation. In future studies, we will explore the effect of excessive activation of NFAT1 in GBM cells.

The discovery that NFAT1 regulates invasion, but not proliferation, in GBM suggests that it may play a role in GBM recurrence. Typically, chemotherapeutic agents impact the cell cycle and primarily affect highly proliferative cells. According to our results, NFAT1 may not correlate with chemo-sensitivity and therefore cannot be used as a prognostic indicator for chemotherapy. This notion is supported by the lack of correlation between NFAT1 expression and survival of GBM patients, who all received standard TMZ chemotherapy (data not shown). On the other hand, our results are grounds for further investigation, since it is widely accepted that NFAT1 regulates genes involved in the immune response. Expressed at elevated levels in an activated form, NFAT1 may play a key role in regulating the microenvironment of GBM, representing a potential leverage point in the treatment of GBM. Although a GBM orthotopic model can be used to examine the role of NFAT1 on the growth of tumors in vivo, it cannot simulate the immune system and the microenvironment of the tumor. Considering the close relationship between NFAT1 and the immune system, we consider this model confusing and unsuitable for our study. Our group is currently engaged in establishing a new animal model to better simulate the *in vivo* environment in future studies.

Given that the immune system plays complex roles in the pathogenesis of malignant tumors such as GBM, regulating the tumor-related immune reaction is critical, and our research may provide some interesting vistas in this direction. One example is CsA, a NFAT signal pathway inhibitor and immunosuppressive drug, which is traditionally associated with an increased risk of neoplasia. However, recent studies provide evidence that CsA (at doses up to 50 mg/kg) does not facilitate tumor progression but partially inhibits tumor growth [36], [37], and the suggestion that CsA could be used in anti-tumor therapy has been emerging. In this study, we showed that in GBM cells, CsA effectively inhibits cell invasion, but has no effect on cell proliferation. CsA therefore has potential as an adjuvant for combined treatment strategies of GBM.

## Conclusion

In this study, we demonstrated for the first time that NFAT1 is overexpressed and activated in GBM. Moreover, we showed that NFAT1 contributes to the invasive phenotype of GBM, but has no effect on cell proliferation. In summary, NFAT1 may play an important role in the malignant progression of GBM, and our study provides a rationale for future research into the use of inhibitors of the NFAT pathway in adjuvant therapy for GBM.
